# Understanding key drivers of performance in the provision of maternal health services in eastern cape, South Africa: a systems analysis using group model building

**DOI:** 10.1186/s12913-018-3726-1

**Published:** 2018-11-29

**Authors:** Martina Lembani, Helen de Pinho, Peter Delobelle, Christina Zarowsky, Thubelihle Mathole, Alastair Ager

**Affiliations:** 1 School of Public Health, University of the Western Cape, Private Bag X17, Bellville, Cape Town, 7535 South Africa; 2 Mailman School of Public Health, Columbia University, New York, USA; 3 University of Montreal Hospital Research Centre and School of Public Health, University of Montreal, Montreal, Canada; 4 Institute for Global Health and Development, Queen Margaret University, Edinburgh, Scotland, UK

**Keywords:** Health systems, Systems dynamics Analysis, Group model building, Maternal health, Quality of care

## Abstract

**Background:**

The Eastern Cape Province reports among the poorest health service indicators in South Africa with some of its districts standing out as worst performing as regards maternal health indicators. To understand key drivers and outcomes of this underperformance and to explore whether a participatory analysis could deepen action-oriented understanding among stakeholders, a study was conducted in one of the chronically poorly performing districts.

**Methods:**

The study used a systems analysis approach to understand the drivers and outcomes affecting maternal health in the district in order to identify key leverage points for addressing the situation. The approach included semi-structured interviews with a total of 24 individuals consisting health system managers at various levels, health facility staff and patients. This was followed by a participatory group model building exercise with 23 key stakeholders to analyze system factors and their interrelationships affecting maternal health in the district using rich pictures and interrelationship diagraphs (IRDs) and finally the development of causal loop diagrams (CLDs).

**Results:**

The stakeholders were able to unpack the complex ways in which factors were interrelated in contributing to poor maternal health performance and identified the feedback loops which resulted in the situation being intractable, suggesting strategies for sustainable improvement. Quality of leadership was shown to have a pervasive influence on overall system performance by linking to numerous factors and feedback loops, including staff motivation and capacity building. Staff motivation was linked to quality of care in turn influencing patient attendance and feeding back into staff motivation through its impact on workload. Without attention to workload, patient waiting times and satisfaction, the impact of improved leadership and staff support on staff competence and attitudes would be diminished.

**Conclusion:**

Understanding the complex interrelationships of factors in the health system is key to identifying workable solutions especially in the context of chronic health systems challenges. Systems modelling using group model building methods can be an efficient means of supporting stakeholders to recognize valuable resources within the context of a dysfunctional system to strengthen systems performance.

## Background

South Africa has of late been faced with almost routine and violent, service delivery protests; difficult labour relations, including in the health sector; widespread concerns about lack of accountability at all levels [1] and a highly politicized environment which affects the daily functioning of public services. In addition, the country faces a quadruple burden of disease, including the world’s largest HIV burden and the largest and most rapidly scaled-up program of anti-retroviral therapy, putting an already frail public health system to the test.

The Eastern Cape is one of South Africa’s nine provinces. Its health outcome and health service indicators are among the country’s worst. Two major reports, by the Treatment Action Campaign and Human Rights Watch, highlighted weak accountability mechanisms in their analyses of health systems failure in the Eastern Cape [2, 3] . While several hospitals, clinics, and individuals are consistently identified as exemplary exceptions, the province’s health system is generally seen as particularly weak and politicized and can be considered to be in a state of “chronic emergency” [2]. Some rural districts stand out as the worst performing among the rest especially in the area of maternal health [4], yet some services and programmes are consistently able to deliver despite the overall weakness and dysfunction of the health system. The Eastern Cape therefore provides a valuable case study to explore health service provision in the context of poor maternal health performance. The aim of this case study was to identify the key drivers and outcomes of maternal health service delivery in one of these poorest performing rural districts in order to strengthen the capacity of district staff to take steps towards reducing maternal mortality. Systems dynamics tools were used to engage stakeholders to analyse the challenges and identify potential solutions. The work was part of a broader study on health systems resilience [5–7], a collaborative project between Mailman School of Public Health, Columbia University and University of the Western Cape‘s School of Public Health. The case study was completed in collaboration with a WOTRO^1^-funded project on, ‘mainstreaming a health systems approach to delivery of maternal health services: transdisciplinary research in Rwanda and South Africa’ (MH-SAR^2^), which implemented participatory action research of health system approaches to improving maternal health in Eastern Cape by researchers from University of the Western Cape, School of Public Health.

### Systems analysis

A variety of forms of systems analysis are increasingly considered as appropriate means to unpacking the complex nature and dynamics of health systems [8]. Systems analysis, which involves a holistic analysis of the complex interrelationships of various parts in the system, offers an effective means of exploring systems determinants of vulnerability and resilience. It is concerned with seeing the ‘whole’ instead of focusing on discrete ‘parts’ [9]. Complex systems are characterized by emergent properties that cannot be explained independently and persist for some time, while adapting to changing conditions [10]. Systems analysis makes it possible for researchers to identify key leverage points within the system. These leverage points are defined as “places within a complex system where a small shift in one thing can produce big changes in everything” [11].

One approach to analysing the dynamics of complex systems is the use of group model building (GMB), which focuses on the creation of causal loop diagrams through intensive, participatory consultation with stakeholders. GMB involves knowledge elicitation from a group of people concerned with a particular issue through group facilitation using scripts that prompt model formulation [12, 13] . GMB has been described as an action research process as it empowers participants by formulating a problem and identifying plausible solutions [14]. Causal loop diagrams (CLDs) are used as a visualisation and communication tool to depict causal relations between selected variables, focusing on positive and negative feedback loops and development trends [8]. According to Videira et al. [15, 16] these types of approaches provide a structured platform for groups of stakeholders from various organisations to participate and engage actively in policy and decision-making processes that foster knowledge co-production and shared learning as well as reinforcement of critical thinking skills.

### Application of systems analysis in the health sector

In recent years, there has been an interest in the application of systems thinking and modelling methods in the health sector owing to its potential to address the inherent complex nature of health systems and public health issues [17, 18] Traditional health technology assessments have been criticised for neglecting the impact of the broader health systems elements crucial for the realisation of intended health system goals [19]. Lebcir [20] argues that the persistent failures in health care systems management are a result of the limitations in “the tools used to analyse, design, and implement actions and policies”, and contests that “managerial decisions are too simplistic to cope with the complexity involved in such system resulting in well intended policies producing unintended consequences” [20]. The author suggests that this misery of policy failures can be minimised by applying a holistic approach to the analysis of health care systems considering the interconnected nature of the system and therefore advocates the use of systems thinking and particularly systems dynamics methodology.

To date a number studies have applied systems analysis in the health sector across the areas of immunization, neonatal mortality, and other health related topics [20–25]. Very few studies have applied systems analysis in the area of maternal health. Sooka and Semwanga [25] and Cramer [26] used forms of this methodology to analyse and model maternal health dynamics in Uganda with respect to the high maternal mortality rates despite efforts made by the Ugandan government. The studies highlighted some useful applications of systems thinking analysis in maternal health care [24] [27].

This study also used systems thinking tools to surface health systems dynamics effecting high maternal mortality rates in the study district. This study however used a qualitative systems analysis aimed at surfacing the structure of the complex system driving poor maternal health outcomes in the district using a participatory approach to engage with stakeholders. The process empowers stakeholders to analyse the problem and understand the system issues affecting maternal health as they develop the systems model and identify key leverage points for intervention.

## Methods

The study used a systems analysis approach that followed a structured participatory method involving four distinct phases: (i) problem analysis and individual interviews; (ii) preliminary data analysis and planning for GMB; (iii) GMB workshop and creation of CLDs; and, (iv) documentation, model refinement and results dissemination. The study was conducted between July 2014 and March 2015.

### Phase 1. Problem analysis and individual interviews

In this phase, the research team and stakeholders at provincial and district level negotiated the focus and boundaries of the case study, which included focusing on maternal health services as compared to looking at the whole health system. The team also agreed to focus on the whole district rather than a part of it. This phase built on contextual knowledge developed through analysing the published and grey literature and a long-standing engagement and collaboration with various actors and institutions through the existing WOTRO funded project. Two staff members from the district were appointed to work with the research team through the rest of the research process as a joint planning team.

After establishing the boundaries, a series of key informant interviews were conducted to solicit information from a broad range of actors, and to familiarize the research team with details of the local context. The key informants were selected depending on their knowledge regarding specific aspects of the system which impact on maternal health. These informants were identified by the district planning team that was selected to work with the research team. Snowballing was also used as the key informants referred the research team to some individuals in the district for specific information. Key informants included the Provincial maternal health manager, two district level managers, two Sub-district health managers, five professional nurses from four health facilities, two hospital chief executive officers, one Sub-district pharmacist, a drug depot manager, four Sub-district emergency transport managers, emergency transport call centre staff, one health promoter and eight patients (two pregnant and six post-partum women) from two rural health facilities and one semi-urban hospital. One patient was interviewed at a rural health clinic; two at a rural health centre and five at the semi-urban hospital.

Patients were randomly selected from a group of women that were present at the health facility at the day of interview. Only staff were given appointment dates and times. For other key informants, the interviews took place in their respective offices. Each participant was provided with information about the purpose of the interview and assured of confidentiality and anonymity as regards participation. An additional letter of clearance was obtained at the district health office to conduct facility interviews. The interviews were conducted by the researchers in English except for those patients who were interviewed in the local language (isiXhosa) using a translator who was employed as a research assistant. Interviews were conducted until a saturation point was reached, whereby there was no new information generated from the various categories of participants [26].

An interview guide was used to structure the discussions with questions focused on health system issues that affect maternal health, including drug supplies, referral systems, transport availability, human resources, leadership, quality of care and other aspects which affect maternal health. The interviews were tape recorded after obtaining permission from the interviewees and notes were taken to capture the main discussion points. The recorded interviews were transcribed by researchers in written scripts. Overall, 24 people were interviewed.

### Phase 2. Preliminary data analysis and planning for GMB

The second phase entailed preliminary data analysis and planning for the GMB workshop. This phase drew together a planning team comprising four researchers and the dedicated two district staff to identify major themes arising from the interviews. Each team member reviewed all the interview transcripts and independently identified key emerging issues, putting each issue on a separate post-it note. The team jointly grouped similar issues together under specific themes. Each theme was then turned into a variable and defined to ensure collective understanding of the meaning of each variable as definitions were based on what the interviewees highlighted as issues surrounding those variables.

**Fig. 1:**
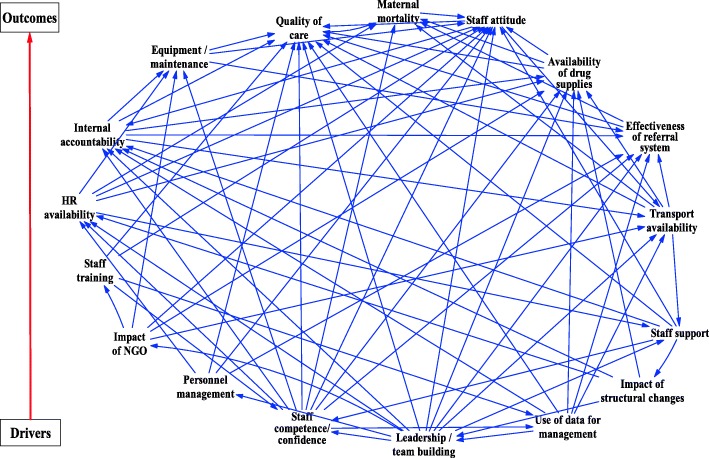
Interrelationship Diagraph

**Fig. 2:**
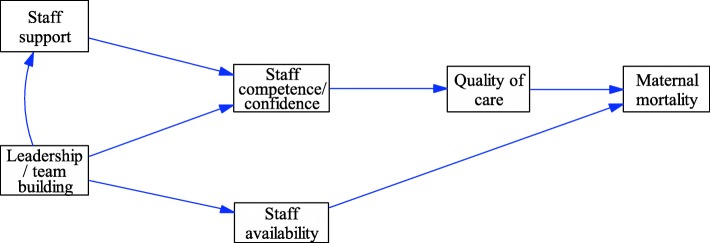
Seed Model

Using these variables, the planning team developed an interrelationship diagraph (IRD) to highlight major links and identified key drivers and outcomes of the maternal health system (Fig. 1). Informed by the IRD the planning team surfaced a ‘seed model’ to be used as a starting point for the model building exercise in the GMB workshop in phase three (Fig. 2). A workshop program was developed featuring “scripts” to structure the stages of discussion [13, 14, 28, 29]. At this stage, the planning team drew a one pager summary providing the research background, aim, objectives and the preliminary findings from key informant interviews. The one pager was used to invite participants to the workshop. This summary was drafted in the form of a letter signed by the District health manager and sent to all stakeholders who were invited to the workshop. The research team requested to have 15 participants invited in order to manage the participatory process well according to expert recommendations [30]. Due to the varied number of stakeholders the district office felt were important to the process, 20 participants were invited, but 23 people turned up as some participants were invited by their colleagues. This clearly shows how committed the district team was in dealing with the issue of maternal health.

### Phase 3. Group model building (GMB) workshop and creation of CLDs

The third phase involved the GMB workshop itself, a 1-day session with 23 participants selected by the district planning team to represent a spectrum of stakeholders providing key perspectives on the functioning of the maternal health care system in the case study district. The participants comprised representatives from the Provincial Department of Health, district and sub-district health offices, hospitals, emergency medical services, and NGO partners.

To ensure success of the GMB process in this diverse group of participants, the facilitator used a series of activities that served to “level the playing field” and challenge their assumptions about the workshop topic. Participants were subsequently asked to sketch the situation of pregnant women in the district using pictures and a few words, bringing in multiple perspectives as represented by the attending stakeholders. This exercise, referred to as the production of “rich pictures”, generated a lot of discussion and connected with many of the issues that had been covered in prior interviews. At this point the research team presented the main issues emanating from the key informant interviews to the workshop participants and requested their comments, additions or subtraction. This process provided an opportunity to reach consensus on key variables, adding new variables that were missed in the initial analysis, and acted as a triangulation process for the information collected from the key informants.

After agreeing on the key issues /variables identified from key informant interviews and additional variables drawn from the “rich picture” discussions, the next step involved participants drafting an IRD to surface interlinkages and potential causal relationships among the identified system variables. Participants were divided into three small groups to explore the relationships among these variables. This exercise generated a lot of discussion among stakeholders and served to facilitate a shared understanding of some of the complex dynamics shaping the operation of the health system.

**Fig. 3:**
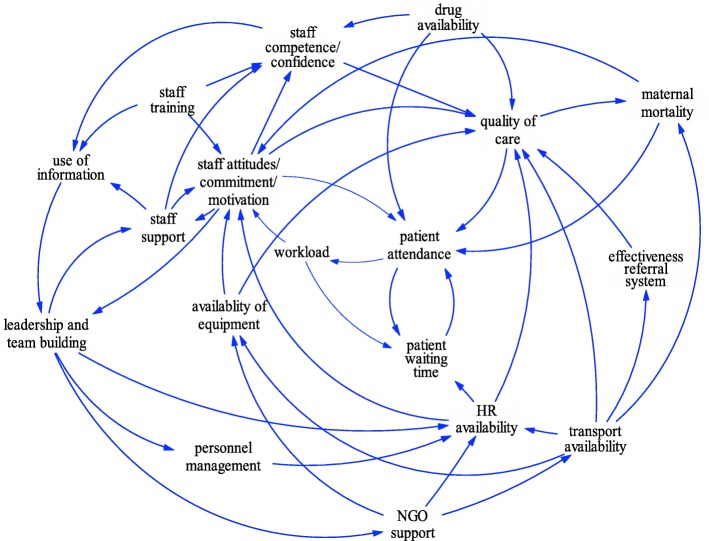
Preliminary Causal Loop Diagram representing factors influencing maternal mortality in OR Tambo District developed during group model building

The next step in this phase was to develop casual loop diagrams. This was also done in small groups. Participants were first presented with the ‘seed model’ (Fig. 2) that was created earlier by the planning team to use as a starting point for the development of causal loop diagrams (CLDs). The seed model was first confirmed by participants and each group started to develop their own CLD from this seed model using the IRD and rich pictures as reference points. Each group developed a CLD that reflected the systems structure driving maternal health outcomes in the case study district. These CLDs were captured by research team members using Vensim PLE software.^3^ CLDs from each group were consolidated into a single model (Fig. 3).

The GMB session ended by having participants reflect on strategies to address the situation revealed through the systems analysis and begin to identify some key leverage points for implementation of system changes.

### Phase 4. Documentation, model refinement and dissemination of study findings

The fourth phase involved documentation, model refinement and dissemination of study findings. Model refinement involved identification of feedback loops and key leverage points in the system by the research team. The research team also drafted a study report that was sent to the district planning team for comments. A feedback workshop was organized to share and discuss the systems model with a broader audience in the district for validation and seek input from those that were not present during the GMB workshop. The model was discussed in small groups and served as platform for the development of local action plans, informed by the presentation of best practices in other Provinces and commitment planning amongst workshop participants. The outcomes and some of the action plans developed are being implemented in the ongoing WOTRO Action Research work.

### Study limitations

For an in-depth analysis using GMB methodology, a 2–3 days workshop process is recommended [12]. Due to time constraints on the part of stakeholders, however, the workshop needed to limited to one full day. This means that there was less time available to elaborate all potential relationships among the factors of the full systems model. Similarly, although the number key informant interviews conducted was seen to achieve saturation of elicited constructs, it is possible that broader consultation across the sub-districts may have highlighted other issues. Finally, while development of causal loop models to represent shared understanding of systems functioning is recognised as an appropriate end-point of systems analysis [12, 14], quantitative validation of models is a common feature of systems dynamics approaches [10, 12]. However, in this context, HMIS and other data sources were inadequate to pursue sensitive analyses and validation.

## Results

Eighteen variables were confirmed as key drivers or outcomes of maternal health in the district by stakeholders during the GMB session. Most of the variables were identified through informant interviews while a few were added through discussion among stakeholders during the workshop. Further confirmation of these variables was done with a bigger audience of stakeholders in the district during the dissemination workshop as elaborated in phase 4 of the research process. These variables comprised: quality of care; maternal mortality; patient attendance; patient waiting time; workload; human resource availability; availability of equipment; transport availability; effectiveness of the referral system; staff training; staff attitudes, commitment and motivation; staff competence and confidence; staff support; personnel management; drug availability; leadership and team building; NGO support; and use of information (Fig. 3). Leadership and team building emerged as a key driver of maternal health care in the district, while the key outcome was quality of maternal health services which in turn affect maternal mortality. Each of these key variables is described in the following section.

### Quality of care and maternal mortality

Workshop participants identified a number of factors contributing to quality of care, all of which were considered to be deficient: staff availability, attitudes, commitment and motivation, and competencies; availability of drugs and equipment, and emergency transport services. Late presentation of pregnant women at health facilities and the lack of timely assistance were linked to poor emergency transport, poor services at facility level, poor staff attitudes and long waiting times. One patient observed:

“*For the first baby, we called for an ambulance and it never came and they tried finding the neighborhood car for hire and [it] never came until the time came and I delivered about 3am in the morning at home. Grannies at home and neighborhood adults helped me” (Patient – Rural Health Center)*

### Staff availability and staff attitudes, commitment and motivation

Staff shortages were seen as an enormous challenge in the district. Practically, this meant that midwives perceived frequently having to perform extra duties and work long shifts. This in turn negatively influenced their attitudes, commitment and motivation. Their perception of managers was that managers were indifferent to the welfare of midwives. Managers, in turn, resented having too many programmes to oversee and felt that this workload made it difficult to provide adequate support to staff. One health facility reported:

*“There are only two professional nurses at this health center, the clinic manager has been borrowed to act (in) some position at the sub-District office and she only comes to the clinic a few days a month. We have to attend to management issues in her absence, which increases our work pressure. Our clinic also does not have a medical officer although it is supposed to have two.” (Professional nurse- Rural Health Center)*

Other challenges attributed to staff shortages included the difficulty to attract and retain health workers in the district because of its rural nature and the lack of differentiation in rural allowances across the district. Despite these challenges, some health workers reported to be committed to their work and trying to provide a good service. Most of the committed respondents were elderly, who originated from the district and therefore felt a sense of responsibility to serve their people regardless of the working conditions but also wanting settle in their home district after retirement.

### Staff training, support, and competence

Both GMB participants and key informants reported that, as a result of structural reforms in training and work arrangements, staff competence and confidence had been affected. A growing cadre of nurses working in health facilities was unable to work independently due to lack of experience. The participants argued that the training curriculum had become more theoretical than practical, and nurses were deployed to health facilities without adequate experience.

*“Quality of staff is not to the standard as before due to changes in the curriculum. Before one had to do 3 years (of) general nursing and 1 year midwifery. But now within 4 years, one is doing general nursing, midwifery, and two more specializations” (Chief Executive Officer - Rural Hospital)*

One result of this situation was an increase in referrals for conditions that could have been managed at a lower level of care but were referred because nurses feared being held accountable if something went wrong. Health workers also complained about a lack of support from their managers, leading to demotivation and poor performance.

*“We are working under a lot of frustration without any proper supervision and when the supervisor comes, she is not encouraging us at all” (Nurse - Rural Health Centre)*

### Personnel management

Personnel management was associated with staff availability and retention. Key informants reported that staff recruitment lasted too long, and that there were many acting positions in the district, which lasted for many years. These people felt abused because of being paid a lower salary in spite of working at a higher level. Staff also felt frustrated because of the perceived lack of attention to their concerns.

*“I am resigning because I have been acting in this position for a long time and I have not been given the post. I have asked the district several times but they did not do anything until now and I was told the post has been frozen. I have so many projects to manage and yet I am not compensated for the work I do. I am tired now and I have decided to resign.” (Maternal and child health manager -Sub-district level)*

### Drugs and equipment availability

Many interviewees complained about a shortage of drugs and equipment, although this was often due to lack of maintenance rather than absence of the equipment. It was also reported that there was only one technician for the whole district. Broken equipment was usually taken to the dealership, but there was a slow turnaround time due to late payment of government bills.

Pharmacists interviewed in this study also reported staff shortages, including of pharmacy assistants, leading to problems processing orders. This resulted in mistakes that had to be corrected in order to avoid dispatching incorrect orders, which in turn could result in stock-outs at other facilities. Processing of orders in different formats was also adding to the work load in an already under-staffed environment.

*“Our major challenge is human resource shortage at all levels, - clinic, sub district and depot. This affects the proper preparation of orders, screening of the orders and system capturing which may lead to unnecessary delays.” (Sub-district pharmacist)*

### Transport availability

Facilities reported challenges in accessing emergency maternal ambulance services, which was also illustrated in rich picture format during the GMB session, and challenges to attend meetings or conduct supervision. Practices reported by some managers as ways to manage transport challenges included efforts to piggy-back supervision visits onto NGO travel so that NGO vehicles could be used, and sometimes using their own vehicles to visit facilities which were relatively accessible.

*“Some of our facility staff need on-site training that is more like mentoring but there’s no transport to take those people who can do some mentoring to the facilities. If they have to go out, they join … and … when they are going out to do their programs, and they just go to the side where … is going.”(Sub-district manager)*

### Effectiveness of the referral system

Due to perceived poor quality of services, some patients preferred to go directly to a higher level of care, which increased the workload for these facilities. In the case of maternal services, it was reported that staff were overwhelmed as they ended up having to deal with more patients than anticipated. This could result in patients sleeping in the corridor or being left unattended. Lack of transport in remote areas also proved to be troublesome.

Interviews with the emergency transport services and health workers revealed that the turnaround time for certain areas took long due to long distances or bad road conditions. They bemoaned the lack of trained personnel to accompany the patient for assistance in case of emergency. The central booking system at the district call centre also proved ineffective.

*“We have a challenge of delays in transport when we call for emergency because we don’t have a land phone. We use our cellphones which delays in registering at the call center. We have an ambulance here at the clinic but we are not allowed to use it until we get permission from the call center.” (Professional nurse from a rural clinic)*

### Leadership and team building

GMB participants identified leadership as a key driver affecting the health system, noting that well-performing facilities where marked by dedicated leadership and teamwork. Such facilities organized regular meetings and jointly addressed challenges. Their managers supported and were able to retain staff, because the latter felt motivated and acknowledged, leading to higher levels of confidence and improved quality of care. Figure 3 confirms that GMB participants saw staff availability; staff attitudes, commitment and motivation; staff competence, confidence and empowerment; drug, equipment and transport availability; quality of care; and effectiveness of the referral systems as all influenced by leadership.

### NGO support

NGOs were reported as important actors trying to support the district in a variety of ways, although respondents also expressed some scepticism about NGO accountability and the overall effectiveness of NGO support. Some felt that better arrangements could be made with NGOs to maximize efficiency. Some NGOs also supported the salary payments of health workers, such as health promoters and pharmacists, and their work was often highly appreciated.

*“At least we are motivated by some NGOs who are trying to give us support, they helped us with equipment like a blood pressure machine and stethoscopes” (Clinic manager, semi-urban clinic)*

### Data management and internal accountability

GMB participants reported that data on performance indicators was hardly used to inform evidence-based decision-making, contributing to weak internal accountability. One participant pointed to a lack of interest from managers, and some interviewees reported cases of mismanagement of resources.

### Other issues

Although not included in the causal loop diagrams some broader contextual factors were included in the model development. For example, key informants pointed to structural changes that affected the system performance, including detaching health centres and clinics from hospitals. This denied newly appointed health workers the opportunity to work under supervision of experienced hospital staff before becoming more responsible at their facility.

*“Most of the deaths that occur are classified as avoidable but due to lack of experience the new graduate professional nurses who are isolated in the clinics have no experience to handle certain conditions and they always refer the cases instead of treating the patients” (Manager –District level)*

Another structural change related to the centralization of the drug supply, whereby changes in the procurement process created more challenges due to limited staff capacity. Likewise, the establishment of sub-districts had put more pressure on the health system, because it added another layer of bureaucracy. Power relations and tensions became apparent when sub-district managers felt bypassed in terms of decision-making by their district managers.

## Discussion

The results of the GMB process revealed that the district health team experienced multiple health system challenges that led to poor quality of maternal health services being rendered to their patients. Among these featured poor staff attitudes towards patients, which is a common phenomenon in health facilities in South Africa [31, 32]. The National Department of Health has committed itself to ensure that avoidable maternal, neonatal and under five deaths are averted [33]. However, the progress is still low as noted in this case study.

**Fig. 4:**
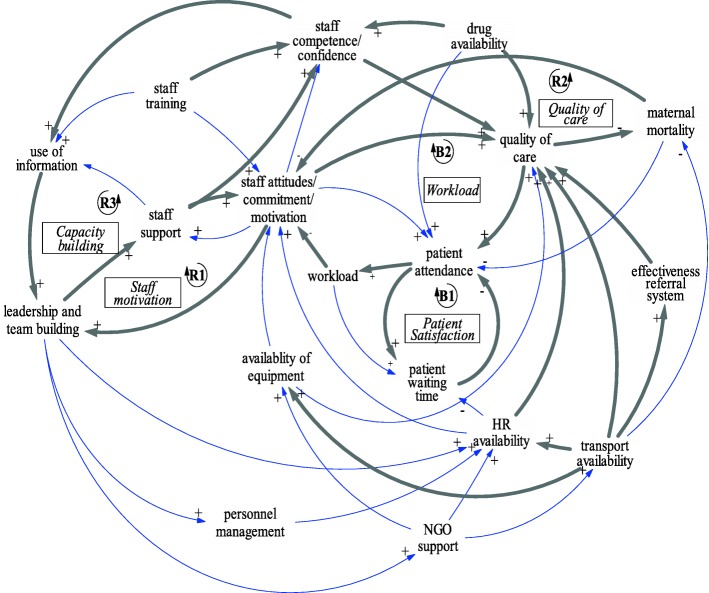
Causal loop diagram depicting key pathways of influence on maternal services within OR Tambo District

Although CLDs only capture the structure of the system, and not necessarily the strength of the relationships between variables, systems mapping and CLDs were used by stakeholders in this study to begin to unpack the ways in which multiple factors were connected in contributing to this poor performance. In doing so they could identify feedback loops (Fig. 4) that both resulted in the performance being intractable to address and suggest strategies for sustainable improvement [34]. For example, quality of leadership was shown to have a pervasive influence on the overall performance of the system with links to several other factors and feedback loops,such as staff motivation *(reinforcing feedback loop R1)* and capacity building *(reinforcing feedback loop R3).* The extent to which *R1* could become a virtuous cycle depends upon its interaction with other feedback loops. Unless workload *(balancing feedback loop B2)* and patient waiting times and satisfaction *(balancing feedback loop B1)* are addressed, improved leadership and support are likely to have limited impact on staff competence and attitudes.

Thus, a virtuous cycle of improved quality of care *(reinforcing feedback loop R2)* requires inputs such as drugs and staff, effective management, including personnel management, and effective referral systems, the latter in turn reflecting the availability of transport, as well as workload *(B2).* In the absence of this concerted attention and action, demand for services might paradoxically be “balanced” in a less positive way: increased workload extends patient waiting times, which could over time lead to reduced patient attendance. With respect to drug availability, despite staffing challenges, the fact that the drug supply depot is within the district facilitated access.

Understanding these dynamics assisted participants to begin to identify strategies to address the factors leading to underperformance of the district maternal care system. Unlike other studies that have used systems dynamics modelling to inform maternal health policies at national level [24, 27], this study aimed at working with stakeholders to analyse and understand the key drivers and outcomes of maternal health and for the stakeholders to identify leverage points to begin to address such factors.

Leadership and team building, staff support and staff competence were identified as key leverage points within their scope of influence and authority to address the chronic underperformance of maternal health services in the district. Participants explored the wider impacts of these leverage points on other variables, including staff support, motivation and commitment. Staff support could also increase staff confidence and competence, which through enhanced use of information, would strengthen leadership and team building. Where leadership and team building failed, the feedback loop became a vicious cycle in which limited support to staff resulted in low motivation and commitment, in turn weakening leadership and team building. It was also observed that where strong leadership existed, the suggested feedback loop dynamics were confirmed: staff in those facilities were reported to be more motivated and improved health outcomes were noted.

Sooka and Rwashana [25] in their interviews with mothers attending antenatal care, nurses, and administrators, reveal similar issues as in this study although expressed using slightly different terminology. These include long distance to the hospital, insufficient hospital facilities such as beds, shortage of staff, inadequate obstetric facilities and staff work load. They use two CLDs, one that focuses on demand for maternal healthcare services and the other on the general maternal health care sub-system. The CLD on demand for maternal health care services consist of factors such as accessibility, number of reproductive females in the population, level of trust in the system driven by effectiveness of the maternal health care system, affordability and level of awareness of maternal health care services. The latter is also influenced by other factors such as the media, beliefs, literacy levels, government actions and poverty levels. The maternal health care subsystem covers factors mainly related to health workers’ motivation in terms of the number of workers that determine workload, salaries and allowance compensation, and safety, skills and training levels, in turn affecting the effectiveness of the maternal health care system alongside other factors in the CLD such as resources that depend on government actions, adoption and usage of technology as well as demand for maternal health care services. The policy recommendations include the need for government to increase funding in the maternal health care services such as construction and maintenance of health facilities, recruitment of skilled providers, provision of refresher courses for existing staff, increased remuneration packages, and improvement in emergency obstetric care facilities, including drugs, equipment and supplies. Other recommendations focus on affordability of services, building trust in the health system, increasing awareness on availability of maternal health care services and paying attention to population growth [24]. These recommendations have been found to be effective in addressing maternal health outcomes [35, 36] and have been mentioned by other researchers, including World Health Organisation (WHO) policy guidelines [37].

Key leverage points identified by stakeholders in this study are, as mentioned above: leadership and team building, staff support and staff competence. Participants identified other factors requiring attention, including *effective collaboration with NGOs (*a dimension of teamwork which reflects effective leadership)*, resource availability in terms of human resources, drug supplies, and equipment* (both a critical input and reflection of the effectiveness of leadership in securing other essential inputs), and *use of information*. Valuable information is generated to guide managers in their work priorities, but rarely meaningfully used. Limited availability of human resources at various levels and with various skills was also found to have negative effects on availability of drug supplies, servicing of equipment and staff workload leading to poor quality of care. The need to strengthen *personnel management* functions was hence an area that was identified as necessary to ensure staff retention and quick recruitment procedures.

Participants acknowledged that after going through the systems mapping process prior conceptualisations were challenged and their perception of which factors really affect their health system had changed dramatically. Unlike many health systems in sub-Saharan Africa, they acknowledged that their health system was relatively well funded, and that it had some level of resources to build upon, including equipment which mostly required some maintenance, and drugs, which were available in the central stores but lacked an effective distribution mechanism. Further, participants noted that the lack of staff was not the most critical aspect as had often been portrayed. Rather, the crucial factors were the retention of staff through *mentoring of staff* which could be achieved through *effective leadership and team building*.

Some health facilities had been able to develop good leadership and teamwork, but this was largely absent. Few managers had strong leadership capacity, and it was therefore recognized that the district was responsible to identify people with leadership skills and put them in strategic places to ensure benefits to the district as a whole. Leadership capacity has been identified as a key constraint to effective health service delivery in South Africa [38], and there has been much interest in leadership capacity development recently, considering health systems as complex adaptive systems that require effective leadership to deal with such complexities unlike managers who are only concerned with “doing things right” [39, 40].

Identifying and relocating people with adequate leadership skills was a necessary but not sufficient long term strategy, however. The impact of workload (B2) and patient satisfaction (B1) balancing loops is such that the system will continue to generate resistance to change - and restrict achievement of the goals of improved quality of care and reduced maternal mortality - unless attention is paid to workload and waiting times. Where strong leadership existed within the district, this was achieved through improved personnel management and NGO support.

## Conclusion

Understanding the complex interrelationships of factors in the health system is key to identifying workable solutions especially in chronic underperforming systems. Systems analysis using group model building – and in particular the identification of feedback loops linking various factors – served to highlight leverage points for intervention. Further, producing such analysis through a participative group modelling process that engaged a broad range of stakeholders served to provide the basis for a shared understanding of the problem and identification of potential solutions. Securing a means for collaborative analysis within local health systems facing acute or, as here, chronic adversity is a key step towards bolstering health systems resilience. Resilience requires systems assets to be recognized, as well as the patterns of interaction that serve to either build upon or erode such assets. Group model building here provided an efficient means for both: supporting stakeholders in recognizing valuable resources within the context of a dysfunctional system, and identifying systems dynamics – focused around leadership, staff competence and staff support – that provided the most plausible mechanisms to strengthen systems performance. The development of local action plans on the basis of the outputs of the analysis indicates the utility of causal loop analysis in identifying points of leverage for policymakers and service managers. While the qualitative analysis that informed model development did not indicate the strength of influence of relationships between variables, it served to highlight hypotheses of where intervention might be most effectively and feasibly focused. In these circumstances, equipping stakeholders with a shared framework with respect to which their activities and influences are mapped may be the most valuable contribution of this approach.

## Abbreviations

CLD: Casual loop diagram
GMB: Group model building
HIV: Human immune deficiency
HR: Human resource
IRD: Interrelationship diagraph
NGO: Non-Governmental Organisation
UWC: University of the Western Cape
WHO: World Health Organisation

## Footnotes

1 The Netherlands Organisation for Scientific Research

2 Maternal Health Research in South Africa and Rwanda

3 Vensim PLE for Windows Version 6.1 is a simulation software freely available for educational purposes from http://vensim.com/
